# Mode of delivery preferences: the role of childbirth fear among nulliparous women

**DOI:** 10.3389/fpsyg.2023.1221133

**Published:** 2023-11-16

**Authors:** Wafaa Taha Elgzar, Majed Saeed Alshahrani, Heba Abdel-Fatah Ibrahim

**Affiliations:** ^1^Department of Maternity and Childhood Nursing, Nursing College, Najran University, Najran, Saudi Arabia; ^2^Department of Obstetrics and Gynecology, Faculty of Medicine, Najran University, Najran, Saudi Arabia

**Keywords:** mode of delivery, childbirth fear, nulliparous, Cesarean Section, Saudi Arabia

## Abstract

**Introduction:**

The increasing Cesarean Section (CS) rates may be attributed to women’s increasing requests for elective CS. High Fear of Childbirth (FOC), especially among nulliparous women, may be significantly associated with CS preference without medical indications. The current study aims to investigate the impact of childbirth fear on the mode of delivery preference among nulliparous women.

**Methods:**

A cross-sectional correlational study was performed in the Maternal and Children Hospital (MCH) from the beginning of October 2022 to the end of February 2023 and incorporated a convenience sample of 342 nulliparous women. The data was collected using a self-reported questionnaire comprising participants’ demographic and obstetrics characteristics and the FOC questionnaire. A logistic regression model examined the relationship between CS preference and the other independent variables.

**Results:**

The results indicated that 74.3% of the nulliparous women preferred vaginal delivery, while 25.7% preferred Cesarean Section. Concerning childbirth-related fear, the highest mean scores were related to fear of clinical procedures, fear of harming or distressing the infant, and fear of pain 5.19 ± 1.13, 5.12 ± 1.27, and 5.09 ± 1.22, respectively. High FOC was present among 74.6%, moderate in 17.3%, and severe in 6.7% of the participants. Logistic regression analysis showed maternal age and monthly income were the significant sociodemographic determinants of choosing CS as the preferred delivery mode (*p* < 0.05). Moreover, the participants who had increased fear of harming or distressing the infant, fear from pain, fear from the body’s ability to give birth, fear from not being involved in decision-making, and overall FOC had a higher probability of choosing CS as the preferred delivery mode compared to the participants who had lower fear (*p* < 0.05).

**Discussion:**

Having high FOC increases the CS preference among nulliparous women. Increased fear of harming or distressing the infant, fear from pain, fear from the body’s ability to give birth, and fear from not being involved in decision- making seem to be significant dimensions of childbirth fear associated with CS preference among nulliparous women.

## 1. Introduction

Pregnancy and childbirth is an important event in a woman’s life. Childbirth is often seen as an important milestone and is a life-affirming event for numerous women. Pregnancy and childbirth are unique experiences for each woman. They can trigger various emotions, such as happiness, hope, anxiety, and fear, making such experiences subjective, multidimensional, and complex (Wigert et al., 2020; Leonard, 2022). It is common for expectant mothers, particularly nulliparous women, to experience varying degrees of anxiety, worries, or fear concerning childbirth (Kananikandeh et al., 2022). Generally, each woman has a varied degree of Fear of Childbirth (FOC); however, the severe form (tocophobia) affects around 1.6%–14% of women (O'Connell et al., 2017; Demsar et al., 2018).

Fear of childbirth is described as a collection of anxious feelings and thoughts related to a woman’s experience of childbirth (Wijma, 2003). Several factors can affect the development of FOC, including biological, social, and psychological. Biological factors include parity, gestational age, pain threshold, and high-risk pregnancy. Social factors involve poor family support, economic problems, and the absence of a spouse. The psychological factors relate to the fear of motherhood, lack of confidence in giving birth, and previous negative experiences (Mohamamdirizi et al., 2018; Imakawa et al., 2022). Other contributing factors include childbirth subjective experiences such as the history of dystocia, instrumental delivery, perineal tears, abuse during a previous birth, uncomfortable delivery room, and many other memories that may significantly impact FOC (Dencker et al., 2019).

FOC can lead to psychological consequences such as anxiety, traumatic stress symptoms, and postpartum depression (Dencker et al., 2019). In addition, FOC may have a relational consequence, especially for expectant fathers and newborns. A recent systemic review reported that FOC among expectant mothers might be reflected to affect 13% of expectant fathers in a pathological and debilitating manner, which can negatively affect their mental health, resulting in stress, anxiety, and depression. The expectant father’s main concern regarding birth is the risk of serious injury or death to the mother or child (Moran et al., 2021). Also, a recent study reported that FOC is significantly associated with the maternal experience of impaired bonding with their newborn, even after controlling sociodemographic factors, contemporary depression, and anxiety disorders. FOC clearly impacts perceived postpartum bonding difficulties but observed mother-infant interaction quality was not affected (Challacombe et al., 2021).

A recent study indicated that nulliparous women experience higher FOC than multiparous women (Hassanzadeh et al., 2020). If such fear is not addressed, it can negatively impact not only the first pregnancy but also subsequent pregnancies, leading to adverse labor outcomes such as prolonged labor, increased labor pain, and the request for epidural analgesia. The decreased blood flow to the pelvic muscles due to increased catecholamines and cortisol levels in the blood caused by fear can lead to hypoxia and intensifying labor pain (Hofberg and Ward, 2003; Gosselin et al., 2016). In addition to the above-mentioned adverse consequences, FOC can contribute to a Cesarean Section (CS) as a preferred mode of delivery. Over the past 20 years, there has been a significant rise in the CS rate in Saudi Arabia. A cross-sectional study conducted at King Abdul-Aziz Medical City, Jeddah, reported that the CS rate was 27% (Alsulami et al., 2020), and another study conducted in Riyadh documented 32.6% (Aljabri et al., 2021). The increasing CS rates may be attributed to the increasing request for elective CS among women (Størksen et al., 2015). The preference for CS is often without medical indications and is primarily driven by FOC (Karlström et al., 2011). Several studies revealed that the main reason for women’s preference for CS is their fear of suffering severe pain during childbirth and lack of knowledge regarding the possible adverse consequences of CS (Nieminen et al., 2009; Torloni et al., 2013; Al-Rifai et al., 2020; Khosravi et al., 2022). Another systemic review by Molgora et al. found physiological fear has no impact on the mode of delivery, but clinically significant fear was associated with an increased rate of emergency CS. However, they added that the cutoff point for clinically significant FOC was unclear among different studies and recommended further research (Molgora et al., 2020). Thus, providing psycho-education to women who experience FOC can have positive outcomes for their present delivery, decrease the need for CS, and improve their expectations of future pregnancies (Fenwick et al., 2015).

Although previous research has demonstrated the relationship between a woman’s FOC and elective CS on maternal request (Ryding et al., 2015; Vaajala et al., 2023), no prior research in the Saudi context has examined the association between childbirth fear and the mode of delivery preference. Therefore, the current study hypothesized that there is a significant impact of FOC on the mode of delivery preference among nulliparous women. The results of this study clarify the ambiguity regarding FOC in Saudi Arabia and provide a strong scientific base for future intervention and research in such areas.

## 2. Subject and methods

### 2.1. Study design and setting

The current study is a cross-sectional correlational study performed in the Maternal and Children Hospital (MCH) affiliated to the Ministry of Health in Najran City, Saudia Arabia. MCH is the only large hospital specializing in maternal and children services in the Najran region; therefore, it serves a large number of the population. The data was collected from the four outpatient clinics that provide antenatal services in MCH hospital.

### 2.2. Study setting and participants

The study incorporated a convenience sample of 342 nulliparous women. The inclusion criteria were nulliparous, free from pregnancy-related complications and diagnosed mental illness (according to the woman’s medical record), singleton pregnancy, the third trimester of pregnancy, aged 18 to 40 years, not advised by a physician for CS, and agreed to participate in the study.

The sample size was calculated based on the Cocharane formula (Uakarn et al., 2021)


$$n=\frac{Z^{2}P\left(1-P\right)}{d^{2}}$$

Where z = 1.96 for a 95% confidence interval; P = cesarean section rate in Saudi Arabia from a previous study (41%; Alabdullah et al., 2021), d is the margin error (0.05). The minimum sample size is 334 pregnant women; after adding 15% to compensate for the anticipated sample loss, the total sample size is 384.

**Figure 1:**
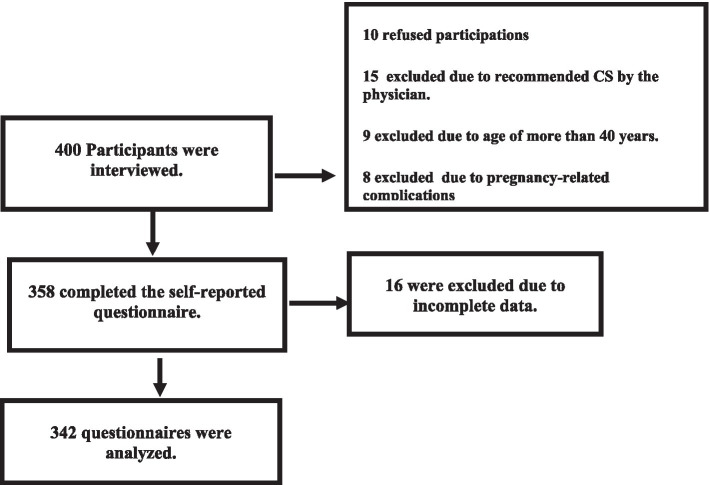
Participants’ flow chart.

The following chart illustrates the participants’ distribution (Figure 1).

### 2.3. Data collection instruments

The data collection instrument is a self-reported questionnaire composed of four parts.

Part I: Participants’ demographic and obstetrics characteristics. This part collects data such as age, residence, occupation, educational level, and satisfaction with monthly income. Obstetric history includes gestational age, regularity of antenatal care (Irregularity of ANC was considered based on whether a participant missed two or more ANC visits), and the number of abortions. Part II: the mode of delivery preference, assessed by one dichotomous question to evaluate whether the woman prefers vaginal birth delivery or CS. Part III is the FOC questionnaire: It was developed by Slade et al. to assess pregnant women’s FOC and composed of 20 items rated on a 4-point Likert scale: strongly disagree (0), disagrees (1), agrees (2), and strongly agree (3). The scoring system was inverted in negative items numbers 1, 3, 5, 8, 10, 14, 17, and 20. The scale was designed to evaluate 10 dimensions related to childbirth fear (each dimension contains two items), namely fear of inability to identify and plan for emergency events, fear of infant harm or distress, fear of pain, fear of the body’s capability to give birth, fear from intranatal or postnatal self-harm, fear from clinical procedures, fear from not being involved in decision making, fear from being alone, fear from the loss of control, and fear from unknown. The total scale score ranged from 0 to 60, with a higher score indicating higher fear. The woman was considered to have low (0–15), moderate (16–30), high (31–45), and severe fear (46–60) based on her score (Slade et al., 2019, 2022). The scale was highly reliable using Cronbach alpha coefficient (r = 0.84; Sanjari et al., 2022).

### 2.4. Data collection procedures

Data collection started from October 2022 to February 2023. The data collection team was present in the antenatal clinic waiting areas from 9 a.m. until 2 p.m. twice weekly for 5 months. Because the study participants were nulliparous women with low-risk pregnancies, randomization was not applicable. Therefore, any participants who fit the inclusion criteria were included in the study using convenience sampling. At the beginning of each data collection session, the data collector explained the study purpose to the participants and then got informed consent. Then, she was given the self-reported questionnaire. The data collection team was present to answer any questions and make required clarifications. If the participant was excluded or refused participation, she was replaced by another one until the required sample size was reached.

### 2.5. Data quality control

The data collection team was composed of two researchers and two data collectors with bachelor’s degrees in nursing and previous experience in data collection. Before data collection, two meetings were held to explain the study proposal, instrument of data collection, research ethics, and data collection procedures. The data collectors discussed any needed clarifications with the researchers. Any questionnaire containing missing data was excluded during the data quality check before data entry to IBM; therefore, 16 questionnaires were excluded from the analysis.

### 2.6. Ethical approvals

The study proposal was approved by the Deanship of Scientific Research and then by the Najran Health Affairs Ethical Committee (IRB Log Number 2022-02 E). Additionally, permission for data collection was granted by the MCH administration. Before each data collection session, the data collector provided a detailed explanation of the study’s purpose and obtained informed consent from each participant. All data collected was anonymous, and participants were assured that their information would remain confidential and that they had the right to refuse participation without any repercussions on their service. The collected data was treated with the utmost confidentiality and used only for research purposes.

### 2.7. Statistical analysis

After completing data collection, data were entered into IBM version 23. The data were described using descriptive statistics such as frequencies, percentages, means, and standard deviation (SD). Among the analyzed data, residence, occupation, level of education, satisfaction with monthly income, regularity of antenatal care, and preferred mode of delivery were categorical variables. While age, gestational age, number of abortions, and FOC were numerical. The total FOC and subscales were obtained by summing items. Binary logistic regression was utilized to determine the CS preference predictors, and the significant level was considered at *p* < 0.05. Before regression, the data was checked for multi-correlations, and the total regression model was checked with the Cox and Snell R Square goodness of fit test.

## 3. Results

Table 1 lists the sociodemographic and obstetrics characteristics of the study participants. The mean age of the participants was 24.58 ± 5.31 years; more than two-thirds (69.9%) of them were aged 20–<35 years. Most of the participants (83.3%) lived in urban areas, and 59.4% were housewives. Approximately three-quarters of the participants had University education and regular antenatal care, 72.5% and 73.7%, respectively. About one-third (33.0%) were moderately satisfied with their monthly income. The mean gestational age was 32.53 ± 2.18 weeks, and the mean number of previous abortions was 0.40 ± 0.78.

**Table 1:** Sociodemographic and obstetrics characteristics of nulliparous women (*n* = 342).

Variables[p]	Total sample	
	*N* = 342	
	*n*	%
**Age (years)**		
<20	42	12.3
20–<35	239	69.9
35–40	61	17.8
Age (years) Mean (SD)	24.58(5.31)	
**Residence**		
Rural	57	16.7
Urban	285	83.3
**Occupational status**		
Employee	139	40.6
Housewife	203	59.4
**Education**		
Read and write	39	11.4
Secondary education	55	16.1
University education	248	72.5
**Monthly income**		
Satisfied	210	61.4
Moderately satisfied	113	33.0
Unsatisfied	19	5.6
**Antenatal care**		
Regular	252	73.7
Irregular	90	26.3
Gestational age (weeks)	32.53 (2.18)	
Mean (SD)		
Minimum	28	
maximum	40	
**Previous abortion**s		
Presence	241	70.5
Absence	101	29.5
Mean (SD)	0.40(0.78)	

Table 2 represents the mean scores of childbirth-related fear among nulliparous women. Concerning childbirth-related fear, the highest scores were related to fear of clinical procedures, fear of harming or distressing the infant, and fear of pain 5.19 ± 1.13, 5.12 ± 1.27, and 5.09 ± 1.22, respectively. Followed by fear of self-harm intra-natal or post-natal4.78 ± 1.49, fear of losing control 3.71 ± 1.10, and fear of unknown 3.52 ± 1.16. The Overall FOC mean score was 39.62 ± 7.46 out of 60.

**Table 2:** FOC mean scores among nulliparous women.

Variables	Minimum	Maximum	Mean	SD
**Domain of FOC**				
Fear from not being able to know and plan for unpredictable events.	0	6	2.54	1.42
Fear of harming or distressing the infant.	1	6	5.12	1.27
Fear from pain.	0	6	5.09	1.22
Fear from the body’s ability to give birth.	0	6	2.77	1.08
Fear from self-harm intra-natal or postnatal.	1	6	4.78	1.49
fear from clinical procedures	0	6	5.19	1.13
Fear of not being involved in decision-making.	0	6	3.45	1.27
Fear from loneliness.	0	6	3.41	1.30
Fear from loss of control.	0	6	3.71	1.10
Fear from the unknown.	0	6	3.52	1.16
Overall FOC	11	60	39.62	7.46

**Figure 2:**
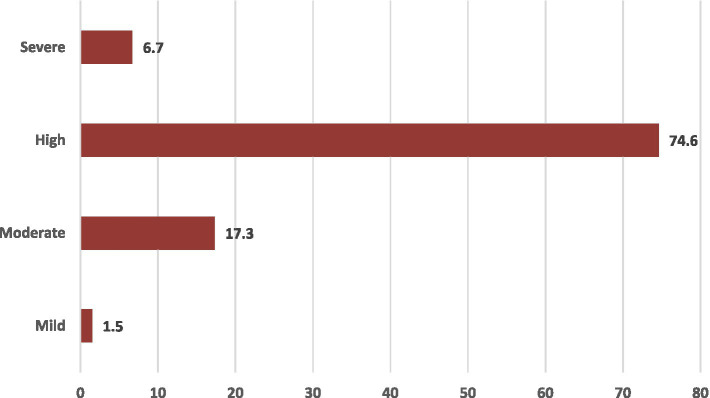
Prevalence of FOC among nulliparous women (*n* = 342).

Figure 2 shows the prevalence of FOC among nulliparous women; high FOC was present in approximately three-quarters of the nulliparous women (74.6%), moderate in 17.3%, and severe fear in 6.7% of the participants.

**Figure 3:**
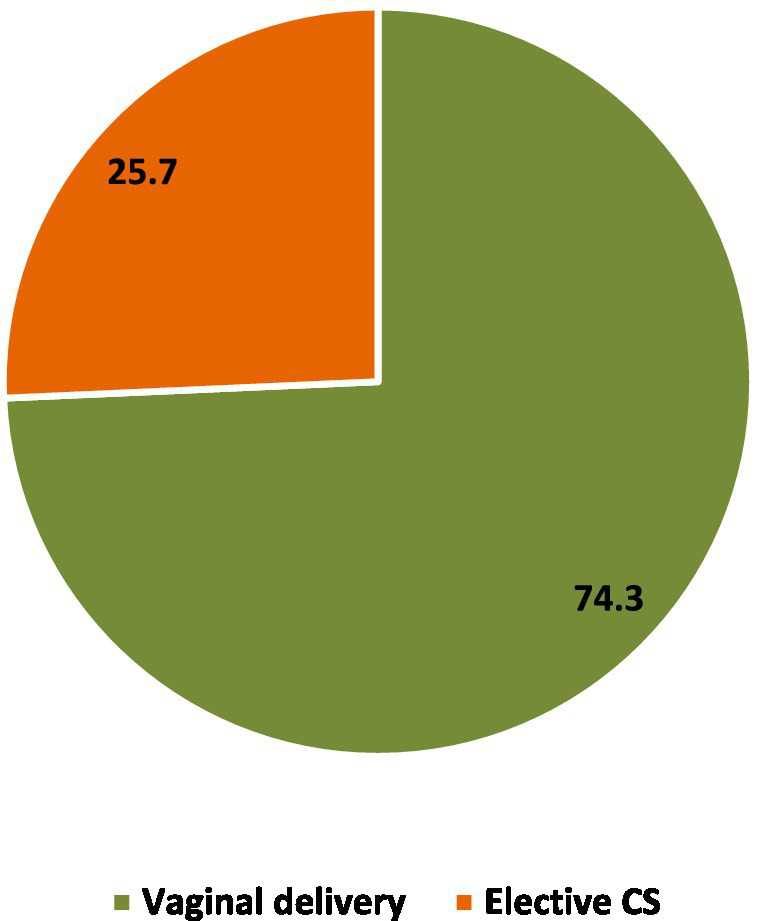
Mode of delivery preference among nulliparous women (*n* = 342).

Figure 3 shows that approximately three-quarters (74.3%) of the nulliparous women preferred vaginal delivery as the mode of childbirth birth, while one-quarter (25.7%) preferred CS.

Logistic regression analysis shows that maternal age and monthly income were the significant sociodemographic determinants of choosing CS as the preferred delivery mode. Women aged between 20–<35 years and those who were 35–40 years had a lower probability of choosing CS as the preferred delivery mode when compared to younger women < 20 years [AOR = 0.165 (0.065–0.421), *p* = 0.000] and [AOR = 0.156 (0.046–0.529), *p* = 0.003], respectively. Similarly, Women with unsatisfied family income [AOR = 0.204 (0.059–0.703), *p* = 0.012] had a lower probability of choosing CS as the preferred delivery mode when taking satisfied family income as a reference (Table 3).

**Table 3:** Logistic regression analysis of sociodemographic and obstetric characteristics associated with choosing CS as the preferred delivery mode.

Predictors	Preferred CS	
	AOR (95% CI)	*p*
Age (years)		0.001^*^
<20	Ref	
20–< 35	0.165 (0.065–0.421)	0.000^**^
35–40	0.156 (0.046–0.529)	0.003^*^
**Residence**		
Rural	Ref	
Urban	0.755 (0.348–1.638)	0.478
**Occupational status**		
Employee	Ref	
Housewife	1.544 (0.824–2.894)	0.176
**Education**		
Read and write	Ref	
Secondary education	1.113 (0.400–3.095)	0.837
University education	0.518 (0.203–1.324)	0.170
Monthly income		0.000^**^
Satisfied	Ref	
Moderately satisfied	0.821 (0.233–2.885)	0.758
Unsatisfied	0.204 (0.059–0.703)	0.012^*^
**Antenatal care**		
Regular	Ref	
Irregular	0.996 (0.524–1.893)	0.991
Gestational age	1.048 (0.919–1.194)	0.484
Number of previous abortion**s**	0.965 (0.688–1.351)	0.834
-2 Log likelihood (326.339)	Cox and Snell R Square (0.170)	Nagelkerke R Square (0.250)

AOR, Adjusted Odd Ratio; CI, Confidence Interval; ^*^significant at *p* < 0.05. ^**^Significant at *p* < 0.001.

As shown in Tables 4, a logistic regression analysis clarified that the participants who had increased fear of harming or distressing the infant, fear from pain, fear from the body’s ability to give birth, and fear from not being involved in decision-making had a higher probability of choosing CS as the preferred delivery mode eight times than the participants who had lower fear, [AOR = 8.369 (1.373–51.275), *p* = 0.029], [AOR = 8.235 (1.255–52.713), *p* = 0.021], [AOR = 8.070 (1.418–50.211), *p* = 0.027], and [AOR = 8.910 (1.398–55.701), *p* = 0.022], respectively. Moreover, the women who had increased fear from the loss of control, fear from the unknown, and overall FOC had a higher probability of choosing CS as the preferred delivery mode than the participants who had lower fear [AOR = 1.395 (1.031–1.945), *p* = 0.033], [AOR = 10.619 (1.724–65.110), *p* = 0.014], and [AOR = 7.402 (1.204–45.706), *p* = 0.031], respectively.

**Table 4:** Logistic regression analysis of FOC domains to CS selection as a preferred delivery mode.

Predictors	Preferred CS	
	AOR (95% CI)	*p*
**Domain of FOC**		
Fear from not being able to know and plan for unpredictable events.	5.250 (0.826–31.567)	0.090
Fear of harming or distressing the infant.	8.369 (1.373–51.275)	0.029^*^
Fear from pain.	8.235 (1.255–52.713)	0.021^*^
Fear from the body’s ability to give birth.	8.070 (1.418–50.211)	0.027^*^
Fear from self-harm intra-natal or postnatal.	4.877 (0.799–30.123)	0.081
fear from clinical procedures	4.768 (0.827–30.158)	0.086
Fear of not being involved in decision-making.	8.910 (1.398–55.701)	0.022^*^
Fear from loneliness.	4.987 (0.812–30.367)	0.079
Fear from loss of control.	1.395 (1.031–1.945)	0.033^*^
Fear from the unknown.	10.619 (1.724–65.110)	0.014^*^
Overall FOC	7.402 (1.204–45.706)	0.031^*^
−2 Log likelihood (334.002)	Cox and Snell R Square (0.238)	Nagelkerke R Square (0.318)

AOR, Adjusted odd ratio; CI, Confidence interval. ^*^Significant at *p* < 0.05. ^**^Significant at *p* < 0.001.

## 4. Discussion

The decision regarding the mode of delivery is difficult and affected by numerous factors. These factors include women’s health and obstetric conditions. In the absence of medical indication, the mode of delivery decision requires months and long discussions to be made and is strongly affected by the woman’s preferences and FOC, especially in nulliparous women (Kjerulff et al., 2019). FOC is usually considered a normal phenomenon because labor is an unpredictable, painful event that may carry positive and negative outcomes. The gold stone to increase maternal preference for vaginal birth is to control FOC at a low or moderate level. The current study is the first one in Saudi Arabia that discussed the role of FOC in determining the mode of delivery preference.

In the present study, the deepest concern of the nulliparous woman regarding FOC was related to fear of clinical procedures, harming or distressing the infant, and pain. In addition, a significant proportion of the women were afraid of self-harm intra-natal or postnatal, fear from the loss of control, and fear of the unknown. High total FOC was present in around three-quarters of the nulliparous women, moderate in 17.3%, and severe fear in only 6.7% of the participants. In the same line, Mortazavi et al. found that FOC was moderate to high among nearly two-thirds of their participants, while severe fear was present among only one-tenth. They added that FOC was strongly associated with old maternal age and lower satisfaction with pregnancy. The major concern regarding FOC among their participants was the loss of control and loneliness, and these two concerns were also present among the current study participants (Mortazavi and Mehrabadi, 2021).

On the contrary (Demsar et al., 2018) reported lower FOC prevalence among women attending prenatal educational classes in Slovenia. They found that moderate FOC was reported among half of their participants, high or very high present among around one quarter, and very severe is reported among only 1.6%. They further elaborated that they distributed their questionnaire after the educational session that explained the progress of labor. It is clearly obvious that educational and prenatal classes significantly decreased FOC. Demsar et al. results magnify the important role that health education may play in making women more familiar with the birth process and consequently decrease FOC (Demsar et al., 2018). However, it seems that women in Slovenia share the same concern regarding FOC, as reported in the current study, as fear of pain, loss of control, fear from clinical procedures, and fear of bodily harm or episiotomy (Demsar et al., 2018). In addition, an Iranian study reported that around three-quarters of their participants had some degree of fear regarding the birth process, and most of them did not attend birth preparation classes. However, they analyzed their data as being afraid or do not be afraid without clarifications about the degree of fear, and this may justify the discrepancy between this study and our study (Khosravi et al., 2022).

Regarding the mode of delivery, one-quarter of the current study participants preferred CS. A similar rate of CS preference was reported in Hong Kong, where 22.9% of their participants considered CS as safer and more controllable compared to vaginal delivery; however, they did not ignore the benefits of vaginal birth (Loke et al., 2015). In Saudi contexts, a recent study investigated the mode of delivery preference among primiparous women. They reported that 13.5% of their participants preferred CS without medical indications, while the vast majority preferred vaginal delivery because of being natural and having a rapid recovery. They further elaborated that the woman’s decision is strongly influenced by her husband’s preference (Alkhazal et al., 2021).

On the contrary, a study conducted in the United Arab Emirates reported that only 9.4% of their participants preferred CS compared to 25% in the current study (Al-Rifai et al., 2020). This discrepancy between the current study and the former may be attributed to the differences in the studies’ sampling and inclusion criteria. The United Arab Emirates study included multiparous and primiparous women in different stages of pregnancy, while the current study incorporated only nulliparous women in the third trimester of pregnancy and more. It is well known from previous literature that nulliparous woman has more FOC, especially during the last trimester, and may ask for CS (Sluijs et al., 2020). Anyway, it is not necessary that all nulliparous women who requested CS will actually deliver using it. A recent Netherlands study found that 29.3% (17 cases) of their nulliparous participant preferred CS, but the actual CS performed for only 6 cases. In addition, nulliparous women who preferred CS had high FOC, which is a significant predictor of performing CS (Sluijs et al., 2020). In addition, prenatal classes and preparation play an important role in reducing CS preference. In Slovenia, a study that evaluated the FOC among women attending antenatal preparation classes reported that only 7.3% of their participants preferred CS (Demsar et al., 2018). These results may shed light on the crucial role that healthcare providers can play in decreasing the CS rate by raising community awareness about possible CS-associated complications. Besides, a higher rate of CS preference was reported in an Iranian study. They reported that near to half of their participant preferred CS and linked this high CS preference to poor knowledge, negative attitude, and high FOC, which again emphasize the important role of healthcare providers in improving the woman’s birth experience (Khosravi et al., 2022).

In the current study, maternal age and monthly income were the significant sociodemographic determinants of choosing CS as the preferred delivery mode. Younger women aged less than 20 years have a higher probability of CS preference when compared to older women. Similarly, Women with an unsatisfied family income had a lower probability of choosing CS as the preferred delivery mode. Young women were reported to prefer CS delivery in the United Arab Emirates because they may have little knowledge regarding the serious CS consequences (Al-Rifai et al., 2020). Therefore, healthcare providers should explain to young women the advantages and disadvantages of each mode of delivery based on their health condition during antenatal care. Furthermore, an Italian study reported that young maternal age is strongly associated with CS preference, while older women mostly preferred vaginal delivery (Torloni et al., 2013). Besides, two recent studies explored the socioeconomic indicators for CS. They found that women with higher family incomes preferred CS as a mode of delivery (Abudoraehem Faisal-Cury et al., 2017; Taye et al., 2021). The justification for this result by Faisal-Cury et al. is that women from higher family incomes may be advised by their doctors to conduct CS in a private setting from which doctors can have illegal payment (Abudoraehem Faisal-Cury et al., 2017). However, in Saudi Arabia, women with higher economic status who can pay for CS may prefer it mainly to avoid pain. In addition, they may perceive CS as a safer delivery mode and have lower complications. The same point of view was reported by Aljabri et al., who reported that 76% of their participants with high family income preferred CS compared to only 5% of women with low family income (Aljabri et al., 2021). Therefore, high CS preference among women with high incomes in Saudi Arabia may be considered a luxury. This finding necessitates increasing community awareness about the complication of unnecessary CS and the advantages of vaginal deliveries, especially among high-income families.

A logistic regression analysis clarified that the participants who had increased fear of harming or distressing the infant, fear from pain, fear from the body’s ability to give birth, and fear from not being involved in decision-making had a higher probability of choosing CS as the preferred delivery mode eight times than the participants who had lower fear. Moreover, the women who had increased fear from the loss of control, fear from the unknown, and overall FOC had a higher probability of choosing CS as the preferred delivery mode than the participants who had lower fear. In Sutherland, Sluijs et al. tried to explain the relationship between FOC and the preferred mode of delivery. They stated that high FOC was associated with CS preference, and women who preferred vaginal birth and delivered with CS had significant postpartum FOC. On the other hand, women who preferred CS and delivered vaginally had low postpartum FOC (Sluijs et al., 2020). Furthermore, Khosravi et al. stated that 85% of women who preferred CS in their study had a considerable level of FOC (Khosravi et al., 2022). Generally, nulliparous women who prefer CS mostly have high FOC, and they may bring their negative thought about vaginal birth with them inside the delivery room. However, it is worth noting that in a previous study, the woman who preferred CS without medical indications had higher actual CS than women who preferred vaginal delivery (Sluijs et al., 2020). Mostly, the nulliparous women bring their negative thoughts and FOC on their delivery day. The actual question now is, can FOC produce an internal physiological reaction that may hinder the delivery process? It is an interesting area of research that requires more investigation and research. If such a relationship is established, FOC will be considered an emergency obstetric condition that requires intensive management. Furthermore, Torloni et al. found that 20% of their participants preferred CS due to fear of pain and thinking that CS is less traumatic to the newborn (Torloni et al., 2013). In Pennsylvania, a woman who had a higher level of FOC and anxiety and did not receive childbirth education classes was more likely to prefer CS (Kjerulff et al., 2019). Another study in Hong Kong found that women who preferred CS were more concerned about labor pain, perineal tears, and the safety of the newborn, especially those who got pregnant at an older age (Loke et al., 2015). Finally, FOC seems to be a serious psychological indicator of the women’s choice of delivery mode. In addition, nulliparous women may have more intense fear and, therefore, require more attention from healthcare providers in antenatal preparation. In addition, nulliparous women may perceive CS as a less painful and more comfortable childbirth method and ignore its possible serious complications.

### 4.1. Strengths and limitations of the study

Our study has numerous strengths: It is the first study conducted in Saudi Arabia to investigate the impact of FOC on the mode of delivery preference among nulliparous women. Our study used recent, reliable, and valid instruments to evaluate FOC. The sample size was calculated using a standardized equation with a 95% confidence level. However, the current study did not evaluate some CS-associated factors or previous history of infertility. In addition, the current study randomization was not applicable; therefore, we used a convenience sample.

## 5. Conclusion

High FOC was present in around three-quarters of the participants, and severe fear in 6.7% of the participants. One-quarter of the nulliparous women preferred CS as the mode of childbirth. Having high FOC increases the CS preference among nulliparous women. Increased fear of harming or distressing the infant, fear of pain, fear of the body’s ability to give birth, and fear of not being involved in decision-making seem to be significant dimensions of FOC associated with CS preference. In addition, maternal age and monthly income were the significant sociodemographic determinants of choosing CS as the preferred delivery mode.

## Data availability statement

The raw data supporting the conclusions of this article will be made available by the authors, without undue reservation.

## Ethics statement

The studies involving humans were approved by the Najran Health Affairs Ethical Committee, Najran Health Affairs. The studies were conducted in accordance with the local legislation and institutional requirements. The participants provided their written informed consent to participate in this study. Written informed consent was obtained from the individual(s) for the publication of any potentially identifiable images or data included in this article.

## Author contributions

WE and HI conceived and designed the study. WE, HI, and MA gathered the data. HI conducted the statistical analysis. WE and HI wrote the first draft. WE, HI, and MA contributed essentially with result analysis and in the revision of the manuscript. All authors contributed to the article and approved the submitted version.
